# Supplementary material for: Specific Detection and Enumeration of Burkholderia cepacia Complex by Flow Cytometry Using a Fluorescence-Labeled Oligonucleotide Probe
Source: Microorganisms. 2022 Jun 7;10(6):1170. doi: 10.3390/microorganisms10061170 (PMC9227203; doi:10.3390/microorganisms10061170)
Supplement: Supplementary file 1 [file microorganisms-10-01170-s001.zip › microorganisms-1741339-supplementary-TableS1-FigureS1.pdf]

Table S1. True-positive rate (sensitivity) and true-negative rate (specificity) of flow cytometry assay for BCC.

	No	Strain name	results
BCC	1	*Burkholderia cepacia* PC783	+[a]
	2	*Burkholderia cepacia* AU24442	+
	3	*Burkholderia stabilis* AU23340	+
	4	*Burkholderia pyrrocinia* AU11057	+
	5	*Burkholderia ambifaria* HI2468	+
	6	*Burkholderia anthina* HI2738	± [b]
	7	*Burkholderia metallica* AU0553	+
	8	*Burkholderia metallica* AU16697	± [b]
	9	*Burkholderia contaminans* HI3429	± [b]
	10	*Burkholderia contaminans* AU24637	+
	11	*Burkholderia diffusa* AU1075	± [b]
	12	*Burkholderia arboris* ES0263a	+
	13	*Burkholderia arboris* AU22095	+
	14	*Burkholderia lata* HI4002	+
	15	*Burkholderia cenocepacia* AU1054	+
	16	*Burkholderia cenocepacia* AU0222	+
	17	*Burkholderia cenocepacia* AU19236	+
	18	*Burkholderia cenocepacia* HI2976	+
	19	*Burkholderia cenocepacia* HI2485	+
	20	*Burkholderia cenocepacia* J2315	+
Non-BCC	21	*Burkholderia concitans* AU12121	-
	22	*Burkholderia fungorum* AU18377	-
	23	*Burkholderia fungorum* AU35949	± [c]
	24	*Burkholderia gladioli* AU16341	-
	25	*Burkholderia gladioli* AU26454	-
	26	*Burkholderia gladioli* AU29541	-
	27	*Burkholderia gladioli* AU30473	-
	28	*Burkholderia glumae* AU6208	-
	29	*Burkholderia glumae* AU12450	-
	30	*Burkholderia oklahomensis* ES0634	-
	31	*Burkholderia plantarii* AU9801	-
	32	*Burkholderia plantarii* AU37486	-
	33	*Burkholderia thailandensis* AU13555	-
	34	*Burkholderia thailandensis* AU36262	-
	35	*Burkholderia tropica* AU15822	-
	36	*Burkholderia tropica* AU19944	-
	37	*Caballeronia zhejiangensis* AU10475	-
	38	*Caballeronia zhejiangensis* AU12096	± [c]

[a]True-positive (+) and true-negative (-)

[b]The average cell number was below 10^3 cells/ml.

[c]The average cell number was above 10^3 cells/ml.

Figure S1. Flow cytometric analysis of BCC through serial gates by the *Kef* probe (A) and TPC assays (B).

(A)

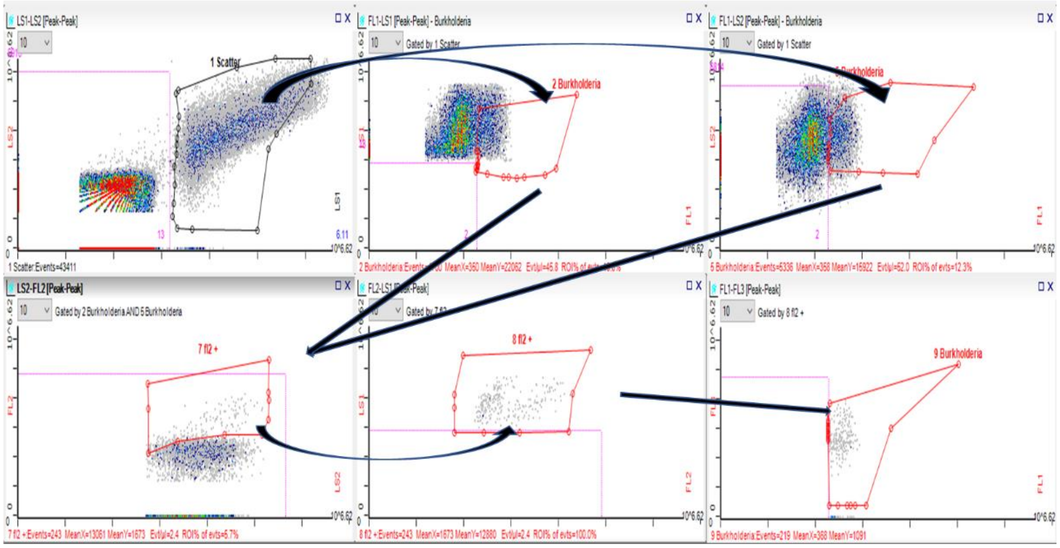

Kef probe assay-Flow cytometric analysis shows the processing of BCC + *Kef* probe through 6 scatter plots. Scatter plot 1) LS1 vs LS2 region 1Scatter data goes to Green vs LS1 (side scatter) and Side scatter vs LS2 (forward scatter = 2Burkholderia and 5Burkholderia regions) which combine to the final region (9Burkholderia) found in the Green vs. Red channel below. The dead cell data goes from the FL1 vs LS1 scatter plot 2Burkholderia region to the Red vs LS2 plot, and from there, to the Red vs LS1 plot below.

(B)

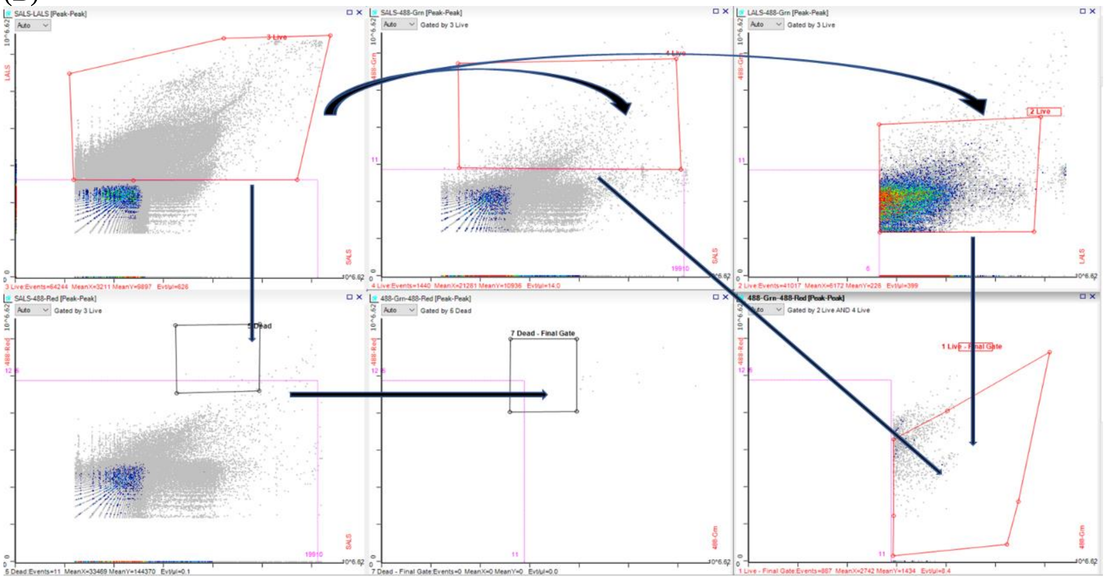

TPC Assay – BCC in phosphate buffered saline + total plate count assay reagent. 1) SALS vs LALS region 3Live data goes to SALS vs Green and Green vs LALS plots (4Live and 2Live regions) which combine to the final region (1Live) found in the Red vs Green Channels plot below. The dead cell data goes from the LALS vs SALS scatter plot 3Live region to the 5Dead region of the SALS vs Red channel plot, and from 5Dead to 7 Dead region of the Red vs Green plot below.
